# Prognostic value of soluble ST2 in AL and TTR cardiac amyloidosis: a multicenter study

**DOI:** 10.3389/fcvm.2023.1179968

**Published:** 2023-08-02

**Authors:** Martin Nicol, Giuseppe Vergaro, Thibaud Damy, Mounira Kharoubi, Mathilde Baudet, Elena Sofia Canuti, Alberto Aimo, Vincenzo Castiglione, Michele Emdin, Bruno Royer, Stephanie Harel, Alain Cohen-Solal, Bertrand Arnulf, Damien Logeart

**Affiliations:** ^1^ Cardiology Department Lariboisière Saint Louis Hospital, University of Paris, Paris, France; ^2^ Cardiology Department, Scuola Superiore Sant’Anna and Fondazione Toscana Gabriele Monasterio, Pisa, Italy; ^3^ Referral Cardiac Amyloidosis Center and Cardiology Department, Mondor Hospital, IMRB U955 and Université Paris Est Créteil all at 94000 Créteil, France; ^4^ Sapienza Universita di Roma, Universita di Bologna, Roma, Italy; ^5^ Immuno-Hematology Department, Saint Louis Hospital, University of Paris, Paris, France

**Keywords:** cardiac amyloidosis, AL, TTR, sST2, biomarker, prognosis

## Abstract

**Background:**

Both light-chain (AL) amyloidosis and transthyretin (ATTR) amyloidosis are types of cardiac amyloidosis (CA) that require accurate prognostic stratification to plan therapeutic strategies and follow-ups. Cardiac biomarkers, e.g., N-terminal pro-B-type natriuretic peptide (NT-proBNP) and high-sensitivity cardiac troponin T (Hs-cTnT), remain the cornerstone of the prognostic assessment. An increased level of soluble suppression of tumorigenesis-2 (sST2) is predictive of adverse events [all-cause death and heart failure (HF) hospitalizations] in patients with HF. This study aimed to evaluate the prognostic value of circulating sST2 levels in AL-CA and ATTR-CA.

**Methods:**

We carried out a multicenter study including 133 patients with AL-CA and 152 patients with ATTR-CA. During an elective outpatient visit for the diagnosis of CA, Mayo Clinic staging [NT-proBNP, Hs-cTnT, differential of free light chains (DFLCs)] and sST2 were assessed for all AL patients. Gillmore staging [including estimated glomerular filtration rate (eGFR), NT-proBNP] and Grogan staging (including NT-proBNP and Hs-cTnT) were assessed for TTR-CA patients.

**Results:**

The median age was 73 years [interquartile range (IQR) 61–81], and 53% were men. The endpoint was the composite of all-cause death or first HF-related hospitalization. The median follow-up was 20 months (IQR 3–34) in AL amyloidosis and 33 months (6–45) in TTR amyloidosis. The primary outcome occurred in 70 (53%) and 99 (65%) of AL and TTR patients, respectively. sST2 levels were higher in patients with AL-CA than in patients with ATTR-CA: 39 ng/L (26–80) vs. 32 ng/L (21–46), *p* < 0.001. In AL-CA, sST2 levels predicted the outcome regardless of the Mayo Clinic score (HR: 2.16, 95% CI: 1.17–3.99, *p* < 0.001). In TTR-CA, sST2 was not predictive of the outcome in multivariate models, including Gillmore staging and Grogan staging (HR: 1.17, CI: 95% 0.77–1.89, *p* = 0.55).

**Conclusion:**

sST2 level is a relevant predictor of death and HF hospitalization in AL cardiac amyloidosis and adds prognostic stratification on top of NT-proBNP, Hs cTnT, and DFLC.

## Introduction

Cardiac involvement is associated with a worse prognosis in patients with amyloidosis. Treatment methods for light-chain (AL) amyloidosis and transthyretin (ATTR) amyloidosis have improved over the last decade, and prognostic stratification should be more and more useful for helping physicians expand therapeutic choices. Cardiac biomarkers are the cornerstone of prognostic assessment (1, 2). In AL amyloidosis, the stratification of patients is based on Mayo Clinic staging (3) that includes cardiac troponins, natriuretic peptides [N-terminal pro-B-type natriuretic peptide (NT-proBNP)], and the differential of free light chains in the revisited Mayo Clinic staging (4). In ATTR amyloidosis, the prognostic score is the Gillmore staging, which includes estimated glomerular filtration rate (eGFR) and NT-proBNP (5), and the Grogan staging, which includes troponin T and NT-proBNP (6). Like most prognostic scores, they are imperfect and could be improved by adding new and relevant variables.

Soluble suppression of tumorigenicity-2 (sST2) is the circulating form of the interleukin-33 membrane receptor released in response to inflammation, fibrosis in various organs, and myocardium stress (7, 8). The prognostic value of sST2 blood levels has been shown in various diseases. In heart failure (HF), sST2 levels add prognostic information on top of natriuretic peptides, and sST2 testing has been included in recent guidelines (9, 10). Inflammation and profibrotic pathways are one of the explanations for cardiac damage in systemic amyloidosis (11), but the prognostic usefulness of the sST2 level measurement has been poorly studied in cardiac amyloidosis (CA), except for one large study that highlighted the prognostic role of sST2 in AL amyloidosis (12).

We aimed to assess the prognostic value of sST2 in CA, compare it with previously validated biomarkers, and assess its added value on top of other biomarkers and Mayo Clinic staging (4), Gillmore staging (5), and Grogan staging (6) for AL and ATTR amyloidosis.

## Methods

### Patient population

This study involved three independent cohorts that included consecutive patients receiving a final diagnosis of CA at Lariboisiere Saint-Louis Hospital (Paris, France, *n* = 105), Henri Mondor Hospital (Creteil, France, *n* = 92), and Fondazione Monasterio (Pisa, Italy, *n* = 103). CA was diagnosed according to the current diagnostic algorithm (13). As part of an elective outpatient visit, all patients underwent clinical examination, blood measurements of high-sensitivity cardiac troponin T (Hs-cTnT), N-terminal pro-B-type natriuretic peptide (NT-proBNP), and free light chains, 12-lead electrocardiography, and comprehensive echocardiographic examination in accordance with the American Society of Echocardiography recommendations (14). Hs-cTnT and NT-proBNP were obtained by commercialized assays (Roche Diagnostics). Serum free light chains were determined using the Freelite assay (Binding Site, Birmingham, Meylan, France). The 2012 Mayo Clinic score (4) was calculated for each patient with NT-proBNP (cutoff 1,800 ng/L), Hs-cTnT (cutoff 40 ng/L) (15), and the differential of plasma free light chains (cutoff 180 mg/L) in AL amyloidosis patients. The 2004 Mayo Clinic staging (3) and stage 3B patients according to Wechalekar et al. (16) were also reported. All AL and TTR amyloidosis patients were included within the first 2 months after the diagnosis. For each TTR patient, Gillmore staging and Grogan staging were calculated. In TTR amyloidosis, prognostic thresholds were 3,000 ng/L for NT-proBNP, 65 ng/L for Hs-cTnT, and 45 mL/min for eGFR according to Gillmore and Gillmore staging (5) and after applying changes in the cTnT cutoff due to the use of new-generation Hs-cTnT (15).

Administration of specific therapies (TTR tetramer stabilizers) at the time of inclusion was checked. Exclusion criteria were age <18 years, pregnant and breastfeeding women, suffering from Randall's disease, and the presence of forms of amyloidosis other than AL-CA or ATTR-CA. The study was carried out according to the principles outlined in the Declaration of Helsinki. Informed and written consent was obtained from all patients.

### sST2 assay

Blood levels of sST2 were determined with the Presage ST2 ELISA (Critical Diagnostics, San Diego, CA, USA) during the diagnosis of cardiac amyloidosis. The assay was performed on an Aspect Reader device with Aspect-Plus ST2 Rapid Test assay cartridges marketed by Critical Diagnostics. The assay principle was based on lateral flow immunofluorescence, and the reproducibility coefficients of variation were between 9% and 20% depending on the concentration level between 75 and 30 ng/L. According to a previous study on amyloidosis, the prognostic threshold was 30 ng/L for sST2 (12).

### Follow-ups and endpoints

Patients were followed up by an exhaustive review of medical files and medical consultations every 3 months and by phone call to referring doctors and patients. When the conclusion of the medical report was unclear, we looked for the use of intravenous diuretics in electronic health records, and hospitalization was considered due to HF only when IV diuretics were prescribed. Follow-up information was retrieved by physicians blinded to sST2 results. The follow-up started at the time of sampling. The endpoint was the composite of all-cause death or first HF-related hospitalization.

### Statistical analysis

Continuous data are expressed as medians and interquartile ranges (IQRs), whereas categorical data are expressed as numbers and percentages. The unpaired *t*-test was used to assess differences in key continuous variables between patients with AL- and ATTR-CA and between patients with or without events. The *χ*^2^ test assessed differences in categorical data between these subgroups. Survival was evaluated with Cox proportional hazards regression analysis, providing estimated hazard ratios (HRs) and Kaplan–Meier curves. The predictive value of most baseline characteristics was explored using Cox regression analysis. To analyze the predictive value of sST2 regardless of Mayo Clinic staging for AL amyloidosis and Gillmore or Grogan staging for TTR amyloidosis, multivariate models included NT-proBNP, cardiac troponin, sST2, and eGFR for TTR amyloidosis and DFLC for AL and the prognostic scores (Mayo Clinic score in AL amyloidosis and Gillmore and Grogan staging in ATTR amyloidosis).

## Results

### Patients and outcomes

The median age was 73 years (IQR 61–81), and 53% were men. Among the 141 patients with AL cardiac amyloidosis, eight were lost to follow-up. Among the 133 AL patients studied, 78 and 28 also had renal and neurological involvement, respectively. Patients with AL amyloidosis received a treatment based on bortezomib, dexamethasone, and cyclophosphamide in 85% of cases and daratumumab in 22% as first-line therapy.

In total, 10% of patients (because of relapse) received IMID-based treatment, and 5% of patients received other treatments (1% of autologous stem cells, 1% of bendamustine because of IgM gammopathy at the diagnosis, 3% of other drugs). Most (around 90%) of AL amyloidosis patients were included at the time of first diagnosis of AL amyloidosis. Of the 133 AL amyloidosis and 152 TTR, 77 were in stage 3 and 35 were in stage 3b, according to the 2004 Mayo Clinic staging (3) and Wechalekar et al. (16).

Among the 159 patients with ATTR-CA, 7 were lost to follow-up and 152 were then studied. Among them, 20 had variant ATTR amyloidosis (15 Val122Ile, 2 Val30Met, 3 other mutations) and 31 received tafamidis. None received other specific therapy.

The median follow-up was 18 months [interquartile range (IQR) 3–34] in AL amyloidosis and 33 (6–45) in TTR amyloidosis. In AL amyloidosis, the primary outcome occurred in 69 (52%) patients, including 30 deaths (17 from cardiovascular causes) and 39 HF hospitalizations. In TTR amyloidosis, the primary outcome occurred in 99 (65%) patients, including 47 deaths (30 from cardiovascular causes) and 53 HF hospitalizations.

The main clinical characteristics of patients according to the type of amyloidosis are presented in Table 1. Patients with AL amyloidosis were younger, had lower systolic blood pressure and lower LV wall thickness, higher LVEF and global longitudinal strain than patients with TTR amyloidosis, and they also had global longitudinal strain, unlike those with TTR amyloidosis. sST2 levels were higher in AL patients than in ATTR patients, while NT-proBNP, cardiac troponin T, and eGFR levels were similar in the two groups.

**Table 1:** Characteristics of patients according to the type of amyloidosis.

	All patients (*n* = 285)	TTR amyloidosis (*n* = 152)	AL amyloidosis (*n* = 133)	*p*
Age, years (median and IQR)	71 (60–80)	73 (61–81)	69 (60–76)	<0.001
Active cancer, *n* (%)	27 (9)	20 (13)	7 (5)	0.07
Autoimmune disease, *n* (%)	23 (8)	19 (13)	4 (3)	0.03
COPD, *n* (%)	15 (5)	13 (9)	2 (2)	0.06
History of HTN, *n* (%)	140 (49)	76 (50)	64 (48)	0.46
Male gender, *n* (%)	195 (68)	114 (75)	81 (61)	0.02
NYHA I–II vs. III–IV, *n* (%)	158 vs. 127	98 vs. 54	60 vs. 73	<0.001
Systolic BP (mmHg)	120 (110–138)	125 (110–140)	118 (109–130)	<0.001
Heart rate, bpm	73 (65–84)	74 (65–82)	81 (71–91)	0.001
Nonsinus rhythm, *n* (%)	46 (16)	30 (20)	16 (12)	0.45
PR duration, ms	180 (154–210)	180 (150–200)	176 (150–200)	0.13
QRS duration, ms	98 (85–128)	112 (86–140)	91 (85–105)	<0.001
IVS thickness, mm	15 (13–18)	16 (13–19)	14 (12–16)	0.04
EDLVD, mm	44 (40–50)	46 (42–50)	42 (38–48)	0.001
LV mass, g/m^2^	149 (115–184)	162 (124–204)	135 (110–168)	<0.001
LVEF, %	55 (45–62)	52 (40–60)	60 (52–64)	<0.001
LA volume, mL/m^2^	45 (37–58)	49 (40–64)	42 (33–52)	<0.001
E/Ea	15 (10–20)	16 (11–20)	15 (10–20)	0.20
Syst PAP, mmHg	41 (31–47)	44 (35–48)	32 (27–35)	0.001
LGS, −%	14.9 (18.1–11)	12.8 (18–9.0)	15.0 (18–12)	0.01
TAPSE, mm	16 (13–20)	16 (13–20)	16 (12–20)	0.37
S wave, cm/s	10 (9–12)	9.0 (7.0–11)	11 (10–13)	<0.001
RV strain, −%	19 (15–23)	18 (8.8–19)	20 (15–23)	0.07
Hemoglobin, g/dL	12.6 (11.4–13.6)	13.3 (12.4–14.3)	12.0 (10.9–13.2)	<0.001
Creatinine, µmol/L	95 (72–130)	87 (71–118)	101 (70–137)	0.21
NT-proBNP, pg/mL	2,381 (616–6,292)	2,640 (455–5,581)	2,183 (645–7,034)	0.19
sST2, ng/L	35 (23–57)	32 (21–46)	39 (26–80)	<0.001
Cardiac troponin T, ng/mL	52 (25–99)	51 (23–88)	55 (27–110)	0.06
eGFR, mL/min	55 (41–78)	62 (43–80)	52 (39–70)	0.07
DFLC, mg/L		0 (0.0–4.0)	50 (24–117)	<0.001
CRP, mg/L	5.0 (4.0–10)	7.0 (4.0–11)	5.0 (3.3–10)	0.35
Corticosteroïds, *n* (%)	16 (5)	2 (1)	5 (3)	0.58
Loop diuretics, *n* (%)	215 (72)	71 (78)	94 (67)	<0.001
ACE inhibitors or AR antagonists, *n* (%)	104 (35)	53 (60)	19 (16)	0.26
MRA antagonists, *n* (%)	87 (29)	35 (31)	38 (27)	0.14
Beta-blockers, *n* (%)	80 (27)	55 (35)	26 (18)	0.24
Pacemaker, *n* (%)	58 (19)	61 (39)	11 (8)	0.70
Defibrillator, *n* (%)	32 (11)	20 (13)	10 (7)	0.007
Anticoagulative therapy, *n* (%)	134 (45)	94 (60)	46 (35)	0.88
Antiplatelet therapy *n* (%)	72 (24)	42 (27)	32 (23)	0.26

BP, blood pressure; CO, cardiac output; E/Ea, peak of pulsed Doppler E wave/average peak of annulus TDI e’ waves; EDLVD, end-diastolic left ventricular diameter; eGFR, estimated glomerular filtration rate; DFLC, differential of free light chains; IVS, interventricular septum thickness; LA, left atrium; LGS, longitudinal global strain; LV, left ventricle; LVEF, left ventricular ejection fraction; RV, right ventricle; Syst PAP, systolic pulmonary arterial pressure; SV, supraventricular; TAPSE, tricuspid annular systolic excursion; COPD, chronic obstructive pulmonary disease; HTN, hypertension; NYHA, New York Heart Association.

The main clinical characteristics of AL and TTR patients according to cardiac events are separately presented in Tables 2 and 3.

**Table 2:** Characteristics of AL patients according to cardiac events.

	All patients (*n* = 133)	Events (*n* = 73)	No event (*n* = 60)	*p*
Age, years	69 (60–76)	69 (67–82)	68 (55–75)	0.78
Active cancer, *n* (%)	7 (5)	4 (5)	3 (5)	0.95
Autoimmune disease, *n* (%)	4 (3)	1 (1)	3 (5)	0.56
COPD, *n* (%)	2 (2)	2 (3)	0 (0)	0.99
NYHA I–II vs. III–IV, *n* (%)	60 vs. 73	29 vs. 44	31 vs. 29	<0.001
Systolic BP (mmHg)	118 (109–130)	115 (106–134)	120 (111–139)	0.04
Heart rate, bpm	81 (71–91)	79 (65–85)	80 (65–83)	0.63
Nonsinus rhythm, *n* (%)	16 (12)	11 (15)	5 (8)	0.76
PR duration, ms	176 (150–200)	180 (160–220)	160 (148–194)	0.02
QRS duration, ms	91 (85–105)	98 (88–140)	89 (82–111)	0.004
IVS thickness, mm	14 (12–16)	14 (13–19)	13 (10–16)	0.002
LV mass, g/m^2^	135 (110–168)	142 (125–196)	126 (89–176)	0.02
LVEF, %	60 (52–64)	55 (40–60)	60 (54–65)	0.03
LA volume, mL/m^2^	42 (33–52)	43 (39–66)	38 (32–50)	0.003
E/Ea	15 (10–20)	16 (12–23)	13 (8.4–17)	0.007
Syst PAP, mmHg	32 (27–35)	40 (34–50)	30 (28–45)	0.002
LGS, −%	15.0 (18–12)	14.0 (9.0–16)	16.3 (20–15)	0.006
TAPSE, mm	16 (12–20)	14 (11–19)	17 (14–20)	0.16
RV strain (−%)	19 (15–23)	16 (13–21)	20 (18–24)	0.03
Hemoglobin, g/dL	11.9 (11.4–13.6)	11.8 (11.3–13.5)	12.0 (11.5–13.7)	0.64
NT-proBNP, pg/mL	2,183 (645–7,034)	4,137 (1,627–8,125)	864 (257–2,471)	<0.001
sST2, ng/L	39 (26–80)	55 (2–72)	30 (18–44)	<0.001
Cardiac troponin T, ng/L	55 (27–110)	70 (42–124)	30 (14–68)	<0.001
eGFR, mL/min/1.73 m^2^	52 (39–70)	49 (38–70)	56 (46–84)	0.29
CRP, mg/L	5.0 (3.3–10)	10 (5.0–11)	5.0 (2.0–10)	0.001
DFLC (mg/L)	50 (24–117)	65 (40–112)	38 (23–56)	0.04
Mayo Clinic staging 1–2 vs. 3–4	61 vs. 72	29 vs. 44	32 vs. 28	0.001
Loop diuretics, *n* (%)	94 (67)	60 (82)	34 (63)	<0.001
ACE inhibitors or ARBs, *n* (%)	19 (14)	10 (14)	9 (17)	0.95
MRA antagonists, *n* (%)	19 (14)	12 (16)	7 (13)	0.14
Beta-blockers, *n* (%)	26 (20)	18 (25)	8 (14)	0.24
Pacemaker, *n* (%)	11 (8)	6 (8)	5 (9)	0.78
Defibrillator, *n* (%)	10 (8)	7 (10)	3 (6)	0.56
Anticoagulant therapy, *n* (%)	46 (35)	22 (30)	24 (44)	0.62
Antiplatelet therapy *n* (%)	32 (24)	20 (27)	12 (22)	0.26

BP, blood pressure; CO, cardiac output; E/Ea, peak of pulsed Doppler E wave/average peak of annulus TDI e’ waves; EDLVD, end-diastolic left ventricular diameter; eGFR, estimated glomerular filtration rate; DFLC, differential of free light chains; IVS, interventricular septum thickness; LA, left atrium; LGS, longitudinal global strain; LV, left ventricle; LVEF, left ventricular ejection fraction; RV, right ventricle; sST2, soluble form of ST2; Syst PAP, systolic pulmonary arterial pressure; SV, supraventricular; TAPSE, tricuspid annular systolic excursion; COPD, chronic obstructive pulmonary disease; NYHA, New York Heart Association.

**Table 3:** Characteristics of TTR patients according to cardiac events.

	All patients (*n* = 152)	Events (*n* = 98)	No event (*n* = 54)	*p*
Age, years	73 (61–81)	76 (67–82)	69 (55–75)	<0.001
Active cancer, *n* (%)	20 (13)	17 (17)	3 (5)	0.04
Autoimmune disease, *n* (%)	19 (13)	16 (16)	3 (5)	0.75
COPD, *n* (%)	13 (9)	8 (8)	5 (9)	0.99
NYHA I–II vs. III–4, *n* (%)	98 vs. 54	47 vs. 51	39 vs. 15	<0.001
Systolic BP (mmHg)	120 (110–138)	120 (106–134)	129 (111–139)	0.35
Heart rate, bpm	73 (65–84)	70 (65–85)	68 (65–83)	0.44
Nonsinus rhythm, *n* (%)	30 (20)	19 (19)	11 (20)	0.99
PR duration, ms	180 (154–210)	120 (160–220)	102 (148–194)	0.03
QRS duration, ms	98 (85–128)	108 (88–140)	90 (82–111)	0.001
IVS thickness, mm	15 (13–18)	18 (13–19)	14 (10–16)	<0.001
LV mass, g/m^2^	149 (115–184)	160 (125–196)	132 (89–176)	<0.001
LVEF, %	55 (45–62)	49 (40–60)	58 (54–65)	<0.001
LA volume, mL/m^2^	45 (37–58)	51 (39–66)	44 (32–50)	0.04
E/Ea	15 (10–20)	17 (12–23)	14 (8.4–17)	<0.001
Syst PAP, mmHg	41 (31–47)	45 (34–50)	41 (28–45)	0.001
LGS, −%	14.9 (18.1–11)	10.0 (9.0–16)	18.3 (20–15)	<0.001
TAPSE, mm	16 (13–20)	15 (11–19)	19 (14–20)	0.002
RV strain (−%)	19 (15–23)	15 (13–21)	20 (18–24)	0.03
Hemoglobin, g/dL	12.6 (11.4–13.6)	12.9 (11.3–13.5)	13.7 (11.5–13.7)	0.002
NT-proBNP, pg/mL	2,640 (455–5,581	3,560 (1,627–8,125)	578 (257–2,471)	<0.001
sST2, ng/L	32 (21–46)	35 (27–72)	27 (18–44)	0.001
Cardiac troponin T, ng/L	51 (23–88)	60 42–124)	26 (14–68)	<0.001
eGFR, mL/min/1.73 m^2^	55 (41–78)	50 (38–70)	72 (46–84)	0.001
CRP, mg/L	5.0 (4.0–10)	9.0 (5.0–11)	5.0 (2.0–10)	0.36
Loop diuretics, *n* (%)	71 (47)	47 (48)	24 (44)	0.92
ACE inhibitors or ARBs, *n* (%)	53 (35)	38 (39)	15 (28)	0.26
MRA antagonists, *n* (%)	35 (23)	27 (28)	8 (15)	0.14
Beta-blockers, *n* (%)	55 (36)	35 (36)	20 (37)	0.78
Pacemaker, *n* (%)	44 (29)	34 (35)	10 (19)	0.04
Defibrillator, *n* (%)	15 (10)	12 (12)	3 (6)	0.006
Anticoagulant therapy, *n* (%)	77 (51)	47 (48)	30 (55)	0.72
Antiplatelet therapy *n* (%)	30 (20)	13 (13)	17 (31)	0.06
Tafamidis	31 (20)	23 (23)	8 (15)	0.15

BP, blood pressure; CO, cardiac output; E/Ea, peak of pulsed Doppler E wave/average peak of annulus TDI e’ waves; EDLVD, end-diastolic left ventricular diameter; eGFR, estimated glomerular filtration rate; DFLC, differential of free light chains; IVS, interventricular septum thickness; LA, left atrium; LGS, longitudinal global strain; LV, left ventricle; LVEF, left ventricular ejection fraction; RV, right ventricle; Syst PAP, systolic pulmonary arterial pressure; SV, supraventricular; TAPSE, tricuspid annular systolic excursion; COPD, chronic obstructive pulmonary disease; NYHA, New York Heart Association.

### sST2 levels: determinants and prognostic values

sST2 levels were higher in patients with AL amyloidosis than in patients with ATTR amyloidosis (Table 1). sST2 levels were significantly higher in patients experiencing adverse events than in patients experiencing TTR-CA and AL-CA (Tables 2, 3). Supplementary Tables S1 and S2 list the predictive values of the most studied predictive variables and of sST2 for the risk of HF hospitalization and/or death in univariate analysis in TTR and AL amyloidosis, respectively.

**Figure 1:**
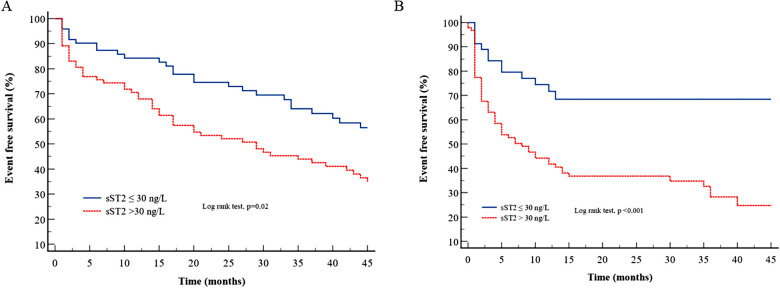
Survival curves for sST2 according to the cutoff of 30 ng/L for the composite endpoint in (**A**) TTR amyloidosis and (**B**) AL amyloidosis.

Table 4 presents the various multivariate Cox models that tested respective predictive values of the variables used in Mayo Clinic or Gillmore or Grogan models. In TTR models (Table 4, Model 1), the sST2 level was not predictive of HF and/or death, and only NT-proBNP had strong and independent predictive value. In AL amyloidosis (Table 4, Model 2), the sST2 level strongly predicted the outcome regardless of the Mayo Clinic score. Even in the most severe patients [defined by NT-proBNP >8,500 ng/L and/or systolic blood pressure <100 mmHg according to Wechelekar et al. (16)], sST2 was still predictive of cardiac events (HR: 2.12, CI: 95% 1.25–3.60, *p* = 0.005). Figure 1 shows the survival curves according to sST2 levels > or <30 ng/L for HF hospitalization and all-cause mortality in TTR and AL amyloidosis.

**Table 4:** Multivariate Cox models for the composite death or heart failure hospitalization in AL and TTR amyloidosis.

Model 1: TTR amyloidosis			
Covariate without sST2	HR	CI 95%	*p*
NT-proBNP >3,000 pg/mL	3.46	1.90–6.30	<0.001
Hs cTnT >65 ng/L	1.63	0.97–2.73	0.07
eGFR <45 mL/min	1.01	0.62–1.66	0.96
AUC 0.71 (95% CI: 0.66–0.76)			
Covariate with sST2	HR	CI 95%	*p*
sST2 >30 ng/L	1.17	0.77–1.89	0.55
NT-proBNP >3,000 pg/mL	4.42	2.48–7.87	<0.001
Hs cTnT >65 ng/L	1.54	0.94–2.50	0.08
eGFR <45 mL/min	1.22	0.70–1.75	0.65
AUC 0.72 (95% CI: 0.68–0.76)			
Covariate with sST2 in Gillmore staging	HR	CI 95%	*p*
sST2 >30 ng/L	1.40	0.93–2.12	0.11
NT-proBNP >3,000 pg/mL	5.03	2.95–8.54	<0.001
eGFR <45 mL/min	1.25	0.79–1.96	0.34
AUC 0.70 (95% CI: 0.66–0.75)			
Covariate with sST2 in Grogan staging	HR	CI 95%	*p*
sST2 >30 ng/L	1.20	0.78–1.84	0.40
NT-proBNP >3,000 pg/mL	4.39	2.55–7.55	<0.001
Hs cTnT >65 ng/L	1.56	0.97–2.53	0.07
AUC 0.72 (95% CI: 0.67–0.76)			
Model 2: AL amyloidosis			
Covariate without sST2	HR	CI 95%	*p*
NT-proBNP >1,800 pg/mL	1.39	0.73–2.64	0.30
Hs cTnT >40 ng/L	3.08	1.47–6.45	0.003
DFLC >180	1.61	0.90–2.90	0.11
AUC 0.69 (95% CI: 0.64–0.75)			
Covariate with sST2	HR	CI 95%	*p*
NT-proBNP >1,800 pg/mL	1.28	0.69–2.38	0.50
sST2 >30 ng/L	3.92	1.65–9.32	0.002
Hs cTnT >40 ng/L	2.48	1.20–5.12	0.01
DFLC >180	1.68	0.94–3.00	0.07
AUC 0.73 (95% CI: 0.67–0.79)			
Covariate with sST2 in Mayo Clinic staging	HR	CI 95%	*p*
2012 Mayo Clinic staging 1–2 vs. 3–4	1.76	1.37–2.26	<0.001
sST2 (ng/L) >30	2.16	1.17–3.99	0.01
AUC 0.70 (95% CI: 0.64–0.75)			

Hs cTnT, high-sensitivity cardiac T troponin; DFLC, differential of free light chains; eGFR, estimated glomerular filtration rate; sST2, soluble form of ST2; AUC, area under the ROC curve.

Interestingly, sST2 was also predictive of cardiac events beyond 1 year of follow-up in AL-CA patients (Supplementary Table S3).

## Discussion

Our study highlights the prognostic value of sST2 in cardiac amyloidosis. Increased sST2 levels are strongly associated with both death and HF hospitalization and remain predictive regardless of other validated predictors, including biomarkers in AL cardiac amyloidosis but not in TTR cardiac amyloidosis.

Prognostic stratification is a major issue regarding the choice of chemotherapy, the monitoring of the patients, and therapeutic intensification in the event of immunochemical and/or organ nonresponse. In ATTR amyloidosis, prognostic stratification could be more and more required because treatment options are rapidly expanding beyond tafamidis (17, 18): TTR silencing by mRNA knockdown or silencing, new TTR stabilizers, and amyloid resorption or extraction. Therefore, finding reliable prognostic markers is essential to identify the patients who will benefit from these new therapies. In TTR and AL cardiac amyloidosis, heart failure is a major issue. HF is predictive of subsequent death in these two populations and could explain almost half of the causes of death. Thus, it makes sense to combine HF and death in the outcome analysis of cardiac amyloidosis and to look for relevant biomarkers that predict both endpoints. In addition, we need biomarkers that could help to define the cardiac response to treatment and that could help to refine therapeutic strategies.

sST2 is the circulating form of the interleukin-33 membrane receptor released in response to vascular congestion and inflammatory or profibrotic stimuli. Increased cardiac expression of ST2 has been observed after cardiac insult (19, 20). By binding IL33, sST2 acts as a decoy receptor and removes the protective effects of IL33 against cardiac hypertrophy, reduction of contractility, and fibrosis (21, 8). Previous studies showed that sST2 predicts outcomes in acute and chronic HF regardless of NT-proBNP, cardiac troponin, and LVEF (22–25). Consequently, sST2 testing has been suggested in the guidelines to refine the assessment of the prognosis of HF patients. In amyloidosis, data on the usefulness of sST2 testing are scarce. Some studies have already assessed the prognostic value of sST2 in AL amyloidosis but not in ATTR amyloidosis. Zhang et al. (26) showed, in 56 AL amyloid patients, that sST2 was a powerful and independent prognostic biomarker for all-cause mortality with a cutoff of 12.3 mg/mL. Kim et al. (27) also showed the prognostic role of sST2 in 73 AL amyloid patients with a median follow-up of 18 months, and the authors suggested that the cutoff value could be 32.6 ng/mL. In a large cohort of 502 AL amyloidosis patients, Dispenzieri et al. (12) showed that sST2 levels >30 ng/mL were independently associated with mortality.

To our knowledge, we present the first data on both AL-CA and ATTR-CA. We report that sST2 levels are higher in AL amyloidosis than in ATTR amyloidosis, even though patients with ATTR amyloidosis exhibited the most severe cardiac remodeling and dysfunction, i.e., greater left ventricular hypertrophy, more altered longitudinal strain, lowered cardiac output, and higher cardiac biomarker levels. sST2 levels were strongly predictive of the outcome irrespective of other cardiac biomarkers and predictors. The cytotoxic effect (28, 29) and the inflammatory changes in cardiomyocytes (30) due to the light chains could explain these differences. Indeed, in AL amyloidosis, light chains polymerize into amyloid deposits that probably induce more severe systemic inflammation than in ATTR amyloidosis, in which there is no underlying neoplasia and where organ damage is more targeted: heart and peripheral nervous systems. Kotecha et al. (31) showed that cardiac AL amyloidosis resulted in greater myocardial edema than ATTR amyloidosis, possibly due to the specific toxicity of the light chains on the myocardium. In addition, edema defined by a native T2 >55 ms was an independent predictor of poor prognosis in AL amyloidosis. sST2 blood levels seem to be a marker of systemic inflammation in the case of neoplasia or certain autoimmune diseases independently of cardiac involvement (32).

Compared with natriuretic peptides and troponin, sST2 blood levels are less influenced by obesity, advanced age, and chronic kidney disease (33). These potential advantages for clinicians were also observed in our study. Indeed, sST2 levels were poorly associated with other biomarkers and renal and LV function. In contrast, NT-proBNP and troponin levels were correlated to each other and renal and LV function.

NT-proBNP levels are strongly correlated with Hs-cTnT levels (data not shown). Of these two biomarkers, cTnT gave the main prognostic information of AL amyloidosis, but it was the opposite in TTR amyloidosis. The increase in cTnT levels is due, at least in part, to the direct myocardial toxicity of light chains in AL amyloidosis, which could be a plausible explanation for the strong predictive value of cTnT levels. In addition, sST2 levels were measured at an early stage in most AL patients before the decrease in light chain levels. On the other hand, the increase in NT-proBNP levels is mainly due to the increase in LV wall stress (mainly related to LV hypertrophy and concentric LV remodeling). LV remodeling was lower in AL vs TTR cardiac amyloidosis. In TTR amyloidosis, the severity of such LV remodeling and its subsequent decreased LV function can lead to most cardiac events.

Our study has some limitations. It is a retrospective study, but we collected data from a heterogeneous population and different centers. All patients with AL amyloidosis had a complete evaluation within 2 months of diagnosis, and some patients received chemotherapy before sST2 measurements, which could impact sST2 levels. sST2 levels could be measured in some patients during HF decompensation, and the predictive value of sST2 can differ according to the delay between decompensation and sST2 measurement (25).

In conclusion, the increase in sST2 blood levels (>30 ng/L) is strongly associated with the risk of death or HF hospitalization in AL cardiac amyloidosis but not in TTR amyloidosis. Further studies are needed to estimate the change of sST2 after treatment in both amyloidosis types.

## Data Availability

The original contributions presented in the study are included in the article/**Supplementary Material**, and further inquiries can be directed to the corresponding author.
