# Supplementary material for: Prognostic value of soluble ST2 in AL and TTR cardiac amyloidosis: a multicenter study
Source: Front Cardiovasc Med. 2023 Aug 2;10:1179968. doi: 10.3389/fcvm.2023.1179968 (PMC10433216; doi:10.3389/fcvm.2023.1179968)
Supplement: Supplementary file 1 [file Table1.docx]

Supplementay Table 1. Univariate Cox analysis for outcome in AL amyloidosis

|  | Composite endpoint | | All cause death | | HF hospitalizations | |
| --- | --- | --- | --- | --- | --- | --- |
|  | HR (CI 95%) | p | HR (CI95%) | p | HR (CI 95%) | p |
| Age | 1.03 (1.01-1.05) | 0.02 | 1.03 (1.01-1.06) | 0.008 | 1.01 (0.98-1.04) | 0.41 |
| NYHA | 1.52 (1.18-1.95) | <0.001 | 1.47 (1.11-1.96) | 0.008 | 1.57 (1.14-2.15) | 0.006 |
| SBP | 0.99 (0.99-1.00) | 0.08 | 0.98 (0.96-0.99) | 0.005 | 0.99 (0.97-1.01) | 0.22 |
| LVEF | 0.97 (0.95-0.97) | 0.01 | 0.98 (0.95-1.00) | 0.06 | 0.97 (0.95-0.99) | 0.03 |
| LGS | 1.08 (1.01-1.15) | 0.02 | 1.06 (0.98-1.15) | 0.12 | 1.11 (1.02-1.19) | 0.01 |
| NT-proBNP> 1800 | 3.03 (1.85-4.95) | <0.001 | 3.98 (2.21-7.16) | <0.001 | 3.94 (2.08-7.46) | <0.001 |
| Hs cTnT > 40 | 4.38 (2.44-7.9) | <0.001 | 3.11 (2.21-5.4) | <0.001 | 5.03 (2.34-11.8) | <0.001 |
| ssT2 > 30 | 2.90 (2.62-5.19) | <0.001 | 2.89 (1.49-5.60) | <0.001 | 3.54 (1.65-7.58) | 0.001 |
| DFLC > 180 | 2.13 (1.22-3.74) | 0.008 | 2.47 (1.34-4.64) | 0.005 | 2.22 (1.11-4.34) | <0.001 |
| Mayo Clinic > 2* | 2.99 (1.83-4.87) | <0.001 | 3.50 (1.98-6.18) | <0.001 | 4.51 (2.33-8.69) | <0.001 |

Hs cTnT: hypersensitive T troponin

dFLC: differential of free light chains

LGS: longitudinal global strain

LVEF: left ventricular ejection fraction

SBP: systolic blood pressure

sST2: soluble form of ST2

*at least 2 among : NTproBNP > 1800, hs TnT > 40, DFLC > 180
