# Supplementary material for: Prognostic value of soluble ST2 in AL and TTR cardiac amyloidosis: a multicenter study
Source: Front Cardiovasc Med. 2023 Aug 2;10:1179968. doi: 10.3389/fcvm.2023.1179968 (PMC10433216; doi:10.3389/fcvm.2023.1179968)
Supplement: Supplementary file 2 [file Table2.docx]

Supplementay Table 2. Univariate Cox analysis for outcome in TTR amyloidosis

|  | Composite endpoint | | All cause death | | HF hospitalizations | |
| --- | --- | --- | --- | --- | --- | --- |
|  | HR (CI 95%) | p | HR (CI95%) | p | HR (CI 95%) | p |
| Age | 1.04 (1.03-1.06) | <0.001 | 1.03 (1.02-1.05) | <0.001 | 1.04 (1.02-1.05) | <0.001 |
| NYHA | 2.02 (1.61-2.55) | <0.001 | 1.85 (1.55-2.22) | <0.001 | 2.11 (1.61-2.74) | <0.001 |
| SBP | 0.99 (0.99-1.00) | 0.06 | 0.99 (0.98-0.99) | 0.009 | 0.99 (0.98-1.00) | 0.01 |
| LVEF | 0.95 (0.94-0.97) | <0.001 | 0.97 (0.96-0.98) | <0.001 | 0.96 (0.94-0.97) | <0.001 |
| LGS | 1.26 (1.10-1.19) | <0.001 | 1.12 (1.07-1.16) | <0.001 | 1.16 (1.10-1.22) | <0.001 |
| NT-proBNP> 3000 | 4.44 (2.88-6.81) | <0.001 | 3.70 (2.63-5.18) | <0.001 | 7.53 (4.47-12.7) | <0.001 |
| Hs cTnT > 65 | 3.42 (2.24-5.20) | <0.001 | 6.78 (4.02-11.4) | <0.001 | 6.92 (4.00-12.0) | <0.001 |
| ssT2 > 30 | 1.62 (1.08-2.42) | 0.02 | 1.84 (1.15-2.94) | 0.02 | 1.92 (1.18-3.13) | 0.01 |
| eGFR <45 | 2.56 (1.70-3.95) | p<0.001 | 0.98 (0.97-0.99) | <0.001 | 0.98 (0.98-0.99) | <0.001 |
| Gillmore >1* | 1.90 (1.50-2.42) | <0.001 | 2.05 (1.54-2.72) | <0.001 | 2.06 (1.52-2.79) | <0.001 |
| Grogan > 1** | 4.71 (2.93-7.57) | <0.001 | 2.47 (1.90-3.21) | <0.001 | 2.54 (1.92-3.37) | <0.001 |

Hs cTnT: hypersensitive T troponin

dFLC: differential of free light chains

eGFR; estimated glomerular filtration rate

IVS: interventricular septum thickness

LGS: longitudinal global strain

LVEF: left ventricular ejection fraction

SBP: systolic blood pressure

sST2: soluble form of ST2

TAPSE: tricuspid annular systolic excursion

*NTproBNP > 3000 or egFR < 45 or both

** NTproBNP > 3000 or Hs TnT > 65 or both
