# Supplementary material for: Prognostic value of soluble ST2 in AL and TTR cardiac amyloidosis: a multicenter study
Source: Front Cardiovasc Med. 2023 Aug 2;10:1179968. doi: 10.3389/fcvm.2023.1179968 (PMC10433216; doi:10.3389/fcvm.2023.1179968)
Supplement: Supplementary file 3 [file Table3.docx]

Supplementary Table 3. Predictive value of variables at 1-year by logistic regression in AL and TTR patients

1. In 60 patients with AL CA (45%) with a follow-up ≥ 12 months

| Variable | Odds ratio | IC 95% | p |
| --- | --- | --- | --- |
| ssT2 >30 | 7.59 | 1.02-78 | 0.04 |
| TnT >40 | 8.04 | 1.12-58 | 0.03 |
| DFLC>180 | 5.07 | 0.52-50 | 0.16 |
| NTproBNP > 1800 | 0.65 | 0.10-4.10 | 0.65 |

1. In 97 patients with TTR CA (64%) with a follow up ≥ 12 months

| Variable | Odds ratio | IC 95% | p |
| --- | --- | --- | --- |
| ssT2 >30 | 1.52 | 060-3.80 | 0.38 |
| TnT >65 | 0.83 | 0.20-3.44 | 0.80 |
| NTproBNP>3000 | 4.17 | 0.97-18 | 0.06 |
| egfr<45 | 2.67 | 0.61-12 | 0.19 |
